# Improved 24-hour urine parameters associated with reduced symptomatic kidney stone recurrence

**DOI:** 10.1007/s00240-025-01910-1

**Published:** 2025-12-22

**Authors:** Wilson Sui, Heiko Yang, Maria C. Escobar, Feres Maalouf, Pablo Suarez, Thomas Chi, Marshall L. Stoller

**Affiliations:** 1 Division of Endourology, Department of Urology, University of Michigan, NCRC Bldg 16, 1 St Floor, #100S-06 2800 Plymouth Road, Ann Arbor, MI 48109 USA; 2 Division of Urology, University of Colorado Anschultz Medical Campus, Aurora, CO USA; 3 Department of Urology, University of California San Francisco, San Francisco, CA USA; 4 Department of Urology, University of Alabama, Birmingham, AL USA

**Keywords:** Urine specimen collection, Urolithiasis, Secondary prevention, Urinary calculi, Nephrolithiasis

## Abstract

On-treatment 24-h urine testing rates are low outside of tertiary care centers. A common criticism is the paucity of evidence demonstrating the optimization of urinary parameters correlates with reduced stone recurrence. Using standard and novel analytic approaches, we sought to evaluate whether improvements in 24-h urine parameters associated with decreased symptomatic stone recurrence. Kidney stone patients who underwent multiple 24-h urine tests and had an initial parameter abnormality were identified from a prospective database. A novel 24-h urine severity score was calculated at both the initial and subsequent tests and used to stratify patients. Negative binomial regression was used to evaluate the impact of analyte changes on stone recurrence. Two hundred patients met inclusion criteria. Low voided volume, hypercalciuria, hyperoxaluria, hypocitraturia and low pH were identified in 56%, 28%, 40%, 49% and 35% respectively. Patients with hypercalciuria and hypocitraturia who consistently normalized these values showed decreased recurrence (p < 0.001). Increasing initial 24-h urine severity scores associated with increased stone recurrence (0.09, 0.18 and 0.31 stone events per year for the lowest, 25-75th and highest quartiles, p < 0.001). On multivariable analysis, severity score improvement or stabilization was associated with reduced risk of recurrence across all quartiles (RRR 0.319 to 0.591, all p < 0.001). Consistent control of 24-h urine parameters was associated with decreased stone recurrence. Not only did novel 24-h urine composite severity scores based on initial 24-h urines independently predict stone recurrence, improvements in this score over time also predicted decreased recurrence.

**Supplementary Information:**

The online version contains supplementary material available at 10.1007/s00240-025-01910-1.

## Introduction

Recurrent nephrolithiasis is frustrating for patients and providers, with estimates of up to 50% recurrence within 5 years of an initial stone event [1–3]. As a disease of morbidity, rarely resulting in mortality, enormous efforts have been devoted to developing and implementing management strategies with the goal of preventing subsequent stone events [4, 5]. Utilization of the 24-h urine test allows for metabolic profile monitoring with targeted dietary or initiation and titration of preventative pharmacologic therapy (PPT).

However, 24-h urine testing, especially repeat testing to monitor for efficacy, is underutilized and adherence to dietary and pharmacologic interventions is modest at best. Only 7% of high risk stone formers undergo any 24-h urine testing [6] and follow up testing amongst those with urinary chemistry abnormalities was as low as 16% [7]. The test is cumbersome for patients to complete and can be challenging for providers to interpret if borderline values or multiple simultaneous abnormalities are present. In addition, though randomized and observational data support the use of PPT, including thiazides [8, 9] and alkali therapy, [10, 11] the value of monitoring analyte changes with repeat 24-h urine collections has not previously been established.

In this context, the Registry for Stones of the Kidney and Ureter (ReSKU) was queried for all patients who attended a metabolic stone clinic and underwent repeat 24-h urine testing. The goal of this study was to evaluate the effectiveness of close metabolic monitoring and management on recurrent kidney stone disease. A secondary objective was to introduce a novel 24-h urine severity scoring system and its utility in identifying patients at risk for recurrent stone disease.

## Methods

### Setting and participants

This was a retrospective cohort study. All adult patients (18 years and older) who underwent 24-h urine testing from 2015 to 2023 and were enrolled in ReSKU were included in this study [12]. The ReSKU registry is a prospectively collected database integrated with the electronic medical record system to gather and store data specifically related to kidney stone disease. All participants in ReSKU provided written consent, and the study received approval from the Committee on Human Research (Protocol 14–14,533). All patients with urinary stone disease are routinely offered enrollment into this prospective database.

## Exposure & predictors

Based on American Urological Association and European Association of Urology guidelines, [4, 5] clinical risk factors for recurrent stone disease were identified and included: 1) recurrent stone former; 2) presence of multiple stones at initial presentation; 3) body mass index (BMI, kg/m^2^) greater than 30; 4) first degree relative with history of nephrolithiasis; and 5) comorbidities associated with nephrolithiasis. The comorbidities included type 2 diabetes, hyperparathyroidism, metabolic syndrome, gout, distal renal tubular acidosis, inflammatory bowel disease, spinal cord injury/paraplegia, bowel diversion and use of a foley catheter or clean intermittent catheterization. The cumulative number of clinical risk factors was then calculated with a maximum score of 5. Receipt of thiazide diuretics or prescription alkali therapy was extracted from the electronic medical record. Patients were excluded if they were already on thiazide or alkali at the time of first 24-h urine collection, or if they started therapy > 365 days from the initial collection. Adherence to therapy was defined as continuous use of medication for at least 180 days from initiation, confirmed by review of the medications section of the electronic medical record. Patients were then categorized as not receiving PPT or as utilizing PPT. Follow up time was defined as time from first urine collection to last urine collection.

Twenty-four-hour urine analytes were abstracted and all studies were performed by a specialized laboratory (Litholink Corporation, Itasca, IL). Twenty-four-hour urine volume, calcium, oxalate, citrate, pH, phosphorus, chloride, sodium, potassium, magnesium, sulfate, urea nitrogen, uric acid, creatinine and creatinine per kilogram were abstracted. The supersaturation index of calcium oxalate (SSCaOx), calcium phosphate (SSCaPhos), and uric acid were reported by Litholink™. Inadequate collections were defined by mg creatinine excretion per kg patient weight (< 11.9 or > 24.4 for males and < 8.7 mg and > 20.3 for females). Only patients who had more than one 24-h urine were included (supplementary Fig. 1). Patients with hypercalciuria (defined below) who were subsequent found to have hyperparathyroidism and underwent surgical intervention were excluded.

The following definitions were used to define 24-h urinary abnormalities: low volume (≤ 2L/day), hypercalciuria (males ≥ 250 mg/day, females ≥ 200 mg/day), hyperoxaluria (> 40 mg/day), hypocitraturia (males ≤ 450 mg/day, females ≤ 550 mg/day), low pH (≤ 5.8) and high pH (≥ 6.2). Patients were categorized by initial 24-h urine abnormality (supplementary Fig. 1) and then subsequent normalization of abnormal analytes was assessed on subsequent collections. To account for variation between tests, patients were categorized based on how consistently they were able to normalize 24-h urine parameters. They were categorized as: “never,” if no subsequent test showed normalized values; as “inconsistent” if 0–50% of subsequent tests showed normalized values; and as “consistent” if 50–100% of subsequent tests showed normalized values.

## Calculation of a novel 24-h urine severity score

A novel methodology which our group has previously described, [13] was used to calculate an overall 24-h urine severity score. An initial severity score was calculated using patients’ initial 24-h urine studies. Changes in the initial 24-h urine abnormalities were used to calculate 24-h urine severity scores at each subsequent collection using the same methods. Patient initial and subsequent 24-h urine scores were then divided into quartiles.

## Outcome

The primary outcome was stone recurrence which was defined as a subsequent stone event > 90 days after initial presentation by patient report of stone passage or procedural intervention (elective or urgent stone treatment). All outcomes were verified by manual review in the electronic medical record.

## Statistical analysis

Analysis of variance was used for continuous variables and chi-square analysis for categorical variables. Negative binomial regression was used to calculate adjusted relative risk ratios. Follow up time was used as an offset variable. First, analyses were by 24-h urinary analyte abnormality to assess for the impact of subsequent 24-h urine normalization consistency of each analyte and stone recurrence. Multivariable negative binomial regression was used to adjust for age, race, gender, cumulative number of clinical risk factors. Second, using the severity scores as described previously, patients were categorized by initial 24-h urine abnormality quartile. The impact of changes in subsequent 24-h urines were then compared using multivariable negative binomial regression adjusting for age, race, gender, cumulative number of risk factors and receipt of PPT. All statistics were performed using SPSS™ v27.

## Results

In total, 200 kidney stone patients met inclusion criteria. Low voided volume, hypercalciuria, hyperoxaluria, hypocitraturia, low pH, high pH, elevated SSCaOx and elevated SSCaPhos were found on initial 24-h urine in 56%, 28%, 40%, 49%, 35%, 44%, 52% and 37% of patients respectively (supplementary Fig. 1). Overall, the demographic characteristics were similar across urinary analyte abnormality groups (Table 1). Mean follow up was 2.3 years.

**Table 1:** Demographic characteristics of the study cohort

		Lithogenic analyte abnormality						Supersaturation abnormality	
	Overall % (n = 200)	Low voided volume % (n = 112)	Hypercalciuria % (n = 56)	Hyperoxaluria % (n = 79)	Hypocitraturia % (n = 98)	Low urine pH % (n = 71)	high urine pH % (n = 87)	Calcium Oxalate % (n = 104)	Calcium Phosphate % (n = 74)
Age (years, mean ± s.d.)	57 ± 15	57 ± 15	57 ± 13	60 ± 13	54 ± 15*	59 ± 15	56 ± 15	56 ± 16	55 ± 15
Sex									
Male	51 (102)	54 (60)	44 (25)	75 (59)**	41 (40)**	55 (39)	51 (44)	56 (58)	53 (39)
Female	49 (98)	46 (52)	55 (31)	25 (20)	59 (58)	45 (32)	49 (43)	44 (46)	47 (35)
Race									
White	62 (124)	65 (73)	68 (38)	63 (50)	49 (48)**	68 (48)	61 (53)	65 (68)	61 (45)
Non-white	38 (76)	35 (39)	32 (18)	37 (29)	51 (50)	32 (23)	39 (34)	35 (36)	39 (29)
BMI									
≤ 30	71 (142)	71 (79)	68 (38)	63 (50)	75 (73)	66 (47)	79 (69)**	72 (75)	76 (56)
> 30	29 (58)	29 (33)	32 (18)	37 (29)	25 (25)	34 (24)	21 (18)	28 (29)	24 (18)
Family history of kidney stones									
No	60 (119)	56 (63)	50 (28)	61 (48)	59 (58)	65 (46)	55 (48)	57 (59)	54 (40)
Yes	40 (81)	44 (49)	50 (28)	39 (31)	41 (40)	35 (25)	45 (39)	43 (45)	46 (34)
First time stone former									
Yes	42 (84)	42 (47)	41 (23)	41 (32)	49 (48)	48 (34)	37 (32)	41 (43)	37 (27)
No—recurrent stone former	58 (116)	58 (65)	59 (33)	59 (47)	51 (50)	52 (37)	63 (55)	59 (61)	64 (47)
Stone multiplicity									
Solitary stone at initial presentation	43 (86)	47 (53)	39 (22)	43 (34)	38 (37)	55 (39)**	32 (28)**	43 (45)	35 (26)
Multiple stones at initial presentation	57 (114)	53 (59)	61 (34)	57 (45)	62 (61)	45 (32)	68 (59)	57 (59)	65 (48)
Comorbidities									
Low risk	73 (145)	74 (83)	73 (41)	62 (49)**	75 (73)	63 (45)**	77 (67)	75 (78)	81 (60)**
High risk	28 (55)	26 (29)	27 (15)	38 (30)	25 (25)	37 (26)	23 (20)	25 (26)	19 (14)

^*^ Denotes p < 0.05 on students' t-test comparing patients with and without urinary analyte abnormality

^**^ Denotes p < 0.05 on chi-square test comparing patients with and without urinary analyte abnormality

Patients who demonstrated hypercalciuria, hyperoxaluria or hypocitraturia on initial collection and then normalized these values consistently on subsequent 24-h urine collections had lower unadjusted recurrence rates (Table 2). After multivariable adjustment, these associations remained significant for patients with initial hypercalciuria and hypocitraturia only (supplementary Fig. 2). For patients with low voided volume, low pH or high pH (Table 2) or abnormal SSCaOx or SSCaPhos on initial collection, normalization of subsequent values did not associate with decreased stone recurrence (supplementary Table 1).

**Table 2:** Association between subsequent 24-h urine analyte normalization and recurrent stone events

	Subsequent 24-h urine normalization	Number of patients	Number of 24-h urines	Initial analyte mean (SD)	Subsequent analyte mean (SD)	Follow up time (person-years)	Stone events per person year	Adjusted Risk Ratio*
Low voided volume (L/day)	Never	53	83	1.3 (0.4)	1.3 (0.4)	109.4	0.2	ref
	Inconsistent	28	107	1.5 (0.3)	2.0 (0.4)	101.7	0.2	2.4 (1.8—3.3)
	Consistent	31	75	1.5 (0.4)	2.6 (0.5)	72.0	0.2	1.1 (0.8—1.5)
Hypercalciuria (mg/day)	Never	21	37	322.0 (110.0)	328.3 (19.1)	38.7	0.4	Ref
	Inconsistent	13	56	302.7 (81.0)	263.8 (15.1)	41.8	0.1	0.2 (0.1—0.4)
	Consistent	21	41	337.4 (121.9)	147.5 (10.5)	43.5	0.1	0.1 (0.1—0.3)
Hyperoxaluria (mg/day)	Never	32	56	62.3 (28.8)	56.9 (14.1)	77.7	0.3	Ref
	Inconsistent	22	85	56.2 (18.6)	44.8 (8.0)	71.3	0.2	0.5 (0.3—0.8)
	Consistent	25	40	51.8 (9.1)	32.2 (6.0)	60.6	0.1	1.30 (0.9—1.9)
Hypocitraturia (mg/day)	Never	55	100	298.9 (132.4)	312.1 (122.5)	104.8	0.3	Ref
	Inconsistent	17	51	304.4 (147.2)	464.6 (98.9)	38.0	0.3	0.8 (0.5—1.2)
	Consistent	25	66	352.1 (139.8)	687.1 (187.9)	60.5	0.1	0.3 (0.2—0.5)
Low pH	Never	16	22	5.4 (0.3)	5.5 (0.2)	43.9	0.1	Ref
	Inconsistent	15	55	5.5 (0.2)	5.9 (0.2)	46.6	0.1	1.2 (0.6—2.3)
	Consistent	38	103	5.6 (0.1)	6.4 (0.4)	84.0	0.2	2.7 (1.6—4.8)
High pH	Never	48	87	6.7 (0.3)	6.7 (0.3)	104.9	0.2	Ref
	Inconsistent	22	76	6.6 (0.3)	6.3 (0.2)	61.1	0.3	1.5 (1.0—2.1)
	Consistent	15	20	6.7 (0.4)	5.8 (0.3)	32.1	0.2	1.4 (0.9—2.3)

^*^ Adjusted model included age, race, gender and cumulative number of clinical risk factors

Of all patients with hypercalciuria, thiazide diuretics were prescribed in 18% of patients, 40% of whom were able to normalize their urinary calcium on a subsequent collection. Similarly, of those patients with hypocitraturia, 27% were prescribed alkali therapy and 50% of these patients were able to normalize their subsequent citrate on at least one subsequent collection. Lastly, of those with low pH, 28% were prescribed alkali, with 74% having at least one subsequent collection with a pH in the normal range or higher.

**Fig. 1:**
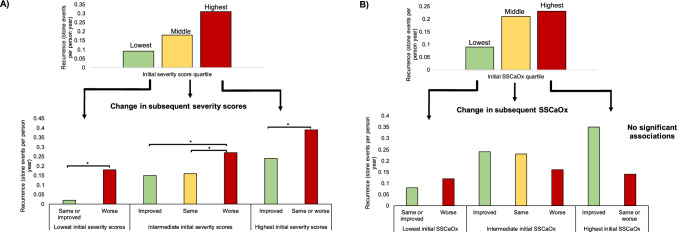
Association between stone recurrence and initial versus subsequent **A** 24-h urine severity scores and **B** supersaturation of calcium oxalate. The 25-50th and 50th-75th quartiles were combined. * denotes statistically significant difference after multivariable adjustment (all p < 0.05)

Based on patients’ initial 24-h urine collections, composite severity scores were tabulated and then divided into quartiles (Fig. 1A). Recurrence rates increased by initial severity score quartile. The impact of subsequent 24-h urine changes was then assessed stratified by the initial severity score. Amongst patients with the lowest initial scores, similar or lower subsequent scores were associated decreased risk of stone recurrence (RRR 0.042, 95% CI 0.016–0.116). For those with initial severity scores in the middle two quartiles, patients with subsequent scores which were in the same or improved quartiles also had lower adjusted risk ratios of recurrence. Lastly for those with the highest initial severity scores, improvement was associated with 0.553 (95% CI 0.371–0.824) adjusted risk ratio for recurrence.

In contrast, the same calculations were performed using supersaturation of calcium oxalate, however, there was no significant association between subsequent change and stone recurrence (Fig. 1B). Complete number at risk, initial and subsequent mean severity scores, follow up time and stone event rate are reported in supplementary Table 2. As a sensitivity analysis, a negative binomial regression was fit using severity scores as continuous variables which showed higher initial (RRR 1.235, 95% CI 1.119–1.363) and subsequent (RRR 1.332, 95% CI 1.195–1.484) severity scores were associated with increased risk of recurrence (supplementary Table 3).

## Discussion

This study has three principal findings. One, consistent normalization of initially abnormal lithogenic urinary analytes – calcium, oxalate, and citrate – on subsequent 24-h urine tests was associated with decreased stone recurrence while normalization of supersaturation of calcium oxalate and calcium phosphate was not. Two, the 24-h urine severity score calculated from the initial 24-h urine can be used to independently predict stone recurrence risk. Third, after controlling for initial 24-h urine severity scores, improvement in subsequent 24-h urine severity scores was also associated with decreased stone recurrence while supersaturation did not. Notably, despite improvements in 24-h urine severity, patients in worse risk categories based on the initial 24-h urine still had the higher stone recurrence risk than those with less severe initial 24-h urines, suggesting a limit to the modifiable component of stone recurrence risk.

The 24-h urine test is challenging for patients to complete, and many healthcare providers struggle with interpreting the results due to the frequent occurrence of multiple abnormalities, the complex interactions between these abnormalities, and uncertain implications of borderline values. Furthermore, stone-formers can have normal 24-h collections and non-stone formers can have abnormal collections. These limitations have contributed to low testing rates, with one study reporting only 7% of high-risk patients undergoing testing [6]. Subsequent testing either to evaluate the progress of dietary modification or to titrate PPT are similarly poor with only 16% receiving repeat testing within 6 months [7]. While there is evidence that stone formers have more severe urinary analytes than non-stone formers, the 24-h urine parameters have not been shown to prognosticate stone recurrence [8, 14–17].

In this context, this study demonstrated not only the value of performing follow up 24-h urine testing but also the importance of aggressive management with the goal of consistently normalizing abnormal urine chemistries. Normalization of hypercalciuria and hypocitraturia was associated with decreases in stone recurrence even after adjustment for demographic and clinical risk factors. Thiazide diuretics were utilized in 18% of those with hypercalciuria and alkali in 27% of those with hypocitraturia and 28% of those with low pH. Otherwise, patients were given comprehensive dietary modification strategies concordant with national guidelines [4, 5]. For example, counseling for all patients with hyperoxaluria involved dietary information on avoidance of oxalate rich foods and for ingesting calcium-rich foods to reduce the bioavailability of intestinal oxalate in the gut and normalization was associated with decreased recurrence [18]. The improvement in patients who were not prescribed PPT was likely due to these behavioral/dietary changes. Our study did not detect an association between voided volume and stone recurrence despite numerous observational [19, 20] and one randomized trial [21] supporting its use. Normalization of voided volume, when other urinary analytes remain abnormal, may not be sufficient to reduce recurrence.

The 24-h urine severity score concept addresses this gap by accounting the breadth and depth of urinary analyte abnormalities in a clinically meaningful way by using standard of care cutoffs. While there is evidence that stone formers have more severe urinary analytes than non-stone formers, there are limited data that 24-h urine parameters predict recurrence [14, 15]. Our group previously reported on the conceptual model and rationale for calculating the severity score from the initial 24-h urine collection and showed that the score independently predicted stone recurrence [13]. This study expands on our previous work, demonstrating that on-treatment testing can identify those whose risk of stone recurrence has not been adequately modified by treatment. Importantly, while we controlled for PPT use, we could not control for changes in dietary therapy as this was inconsistently documented. Nonetheless, our results demonstrate that regardless of how the modifications were accomplished, improving analytes was critical. Additionally, we demonstrated that those with the lowest initial severity scores had the lowest stone recurrence – regardless of changes in subsequent 24-h urines. Similarly, patients with the most significant initial abnormalities also had the worst recurrence rates overall. This suggests that the modifiable component to patient stone recurrence risk may be limited. Genetic predisposition to stone formation, uncaptured clinical, dietary or environmental factors likely contribute to the immutable aspects of stone recurrence risk.

The conceptual model and choice of urinary analytes underlying the severity score is supported by a recent study using data from the Health Professionals Follow-Up Study and Nurses’ Health Studies I and II [22]. This group previously showed the relative risk of being a stone former compared to non-stone formers increased as 24-h urinary parameters escalated with statistically significant differences starting around Litholink™ reference range values [23]. In their recent study, the authors reported a direct linear – or practically linear association between 24-h urinary parameters and stone risk arguing against arbitrary thresholds in clinical use. In addition, on a dominance analysis, urinary calcium, volume, citrate and oxalate were most associated with stone risk – similar to our severity score. Urinary pH did not associate with stone risk in their work which is similar to this study which showed improving abnormal pH did not modify stone recurrence risk. However, there are several important differences, as these studies compared stone formers versus healthy controls whereas our study evaluated stone formers of escalating clinical risk and we included on-treatment studies. Amongst stone formers, it is expected to find smaller differences in 24-h urine parameters yet in spite of this, the severity score was an independent predictor both in the initial and on-treatment setting. Nonetheless, based on this recent study using the HFPS and NHS I/II data, [22] it is clear conceptual model for 24-h urine severity should be further refined. Given the limitations of our modestly sized prospective database, we look forward to continuing this work using larger datasets [24].

There is limited data to suggest changes in urinary supersaturation reflect changes in stone recurrence. In our study, the severity score was able to independently predict stone recurrence at both the initial testing setting and on-treatment while supersaturation did not. This is likely because patients who normalized their supersaturations still had other urinary analyte abnormalities (supplementary Table 1). For example, amongst patients with initially abnormal supersaturation of calcium oxalate, the mean urinary oxalate on subsequent studies ranged 35.1- 42.3 mg/day, suggesting these analytes remained elevated for many patients. Residual abnormalities, uncaptured by supersaturation calculations, explains why the data supporting its use in predicting stone recurrence is limited. In a randomized controlled trial by Borghi et al., patients who developed stone recurrence exhibited no differences in any urinary analyte aside from calcium, including supersaturations [21]. In a subsequent randomized trial by the same group, the group with higher stone recurrence also had lower mean supersaturations of calcium oxalate at all follow up points [25].

This study has several important limitations. First, it is a retrospective study conducted at a tertiary care center, likely involving patients with more severe stone disease and comorbidities, which may limit the generalizability of the results. Although, the data utilized in this study represented detailed clinical, radiographic, and longitudinal patient information not available in large administrative datasets. Second, our outcome measure of stone recurrence includes a composite of stone events – patient reported stone passage and surgical interventions. This measure might be limited by a lack of adjudication and follow-up bias. In addition, patients were not required to be stone free at the start of the observation period, given the modest sample size and inconsistent radiographic follow up both in terms of modality and interval, we did not choose to perform a subset analysis. In our practice, we tailor follow up imaging to the clinical scenario as we do not want to expose stone formers – who are already at higher risk of repeated radiation exposure over time – to the small but undue risk of radiation-related sequelae. Our most common follow up imaging test was renal ultrasound and comparing stone size on renal ultrasound versus computed tomography, or even between ultrasounds, is fraught with inaccuracies therefore we did not choose to include this in our study. However, compared to population-level datasets, this approach may more accurately reflect the patient experience, as spontaneous stone passages are not typically captured. Third, stone composition was not included in the analysis as it was available in a minority of our patients. This reflects contemporary stone management as advancements in flexible ureteroscopy and the widespread adoption of high-powered lasers have also popularized “dusting” techniques reducing stone extraction for analysis. Moreover, stone composition is also known to be variable across laboratories [26] and the relationship between stone composition and 24-h urine studies is inconstant [27, 28]. Although stone composition can complement metabolic counseling, dietary and pharmacologic management in many practices – including our own – is primarily guided by 24-h urine abnormalities. As a result, despite this significant limitation, the findings remain applicable to real-world metabolic stone management.. Fourth, the cohort represented only patients able to closely follow up and complete multiple 24-h urine studies which may introduce selection bias, however, follow up times were similar across each group (supplementary Table 2). In our practice, we routinely conduct follow-up 24-h urine testing at 6- to 12-month intervals after the initial study for all indications, minimizing indication bias. Additionally, the cohort excludes patients who completed only a single study or were lost to follow-up, further reducing bias. Fifth, normalization was included as a binary outcome instead of a continuous outcome such as the magnitude of improvement. Due to the modest sample size, this was not assessed, however, would be an avenue in future work with larger populations. Sixth, patients received on-going advice during the treatment period depending on 24-h urine testing results. There is potential for attrition bias though the number of 24-h urine tests was similar across groups. Lastly, our novel composite severity score has not yet been validated in other institutional or population-level datasets, which we are currently exploring with collaborators.

## Conclusions

While follow up 24-h urine collections is uncommonly undertaken, normalization of initial abnormalities in urinary calcium, oxalate and citrate were associated with improved stone recurrence. A novel overall 24-h urine severity score was an independent predictor of recurrence using both the initial and on-treatment scores. Subsequent worsening of these scores also predicted increased stone recurrence while supersaturation did not suggesting that this scoring system may lead to improved identification of those whose risk of recurrence has not be adequately modified.

## Supplementary Information

Below is the link to the electronic supplementary material.

Supplementary file1 Figure 1. Cohort selection (PNG 52 KB)

Supplementary file2 Figure 2. Impact of 24-hour urine abnormality correction consistency on stone recurrence. *denotes statistically significant difference after multivariable adjustment (all p < 0.05) (PNG 53 KB)

Supplementary file3 (XLSX 12 KB)

Supplementary file4 (XLSX 13 KB)

Supplementary file5 (XLSX 10 KB)

## Data Availability

The data supporting the findings of this study are available in the Registry for Stones of the Kidney and Ureter (ReSKU), a multi-institutional dataset housed at the University of California, San Francisco. Data use agreements are required for access to the data due to availability of patient identifying information (UCSF IRB Protocol 14–14,533). Question regarding access to the data or the code used in the analysis are available by request. Please reach out to Wilson Sui ([wilsonsi@med.umich.edu](mailto:wilsonsi@med.umich.edu)) for further questions or concerns.

## Abbreviations

PPT: Preventative Pharmacologic Therapy
ReSKU: Registry for Stones of the Kidney and Ureter
SSCaOx: Supersaturation of Calcium Oxalate
SSCaPhos: Supersaturation of Calcium Phosphate
