# Supplementary figures and images for: Improved 24-hour urine parameters associated with reduced symptomatic kidney stone recurrence
Source: Urolithiasis. 2025 Dec 22;54(1):8. doi: 10.1007/s00240-025-01910-1 (PMC12722303; doi:10.1007/s00240-025-01910-1)

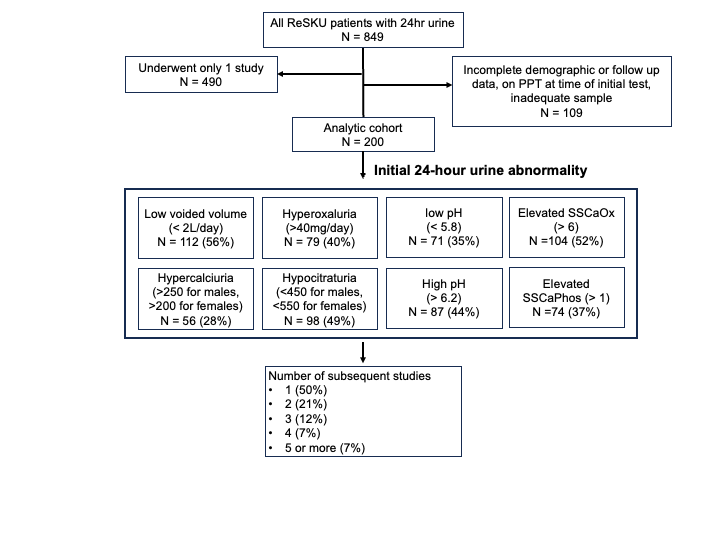

Supplement: Supplementary file 1 — Supplementary file1 Figure 1. Cohort selection (PNG 52 KB) [file 240_2025_1910_MOESM1_ESM.png]

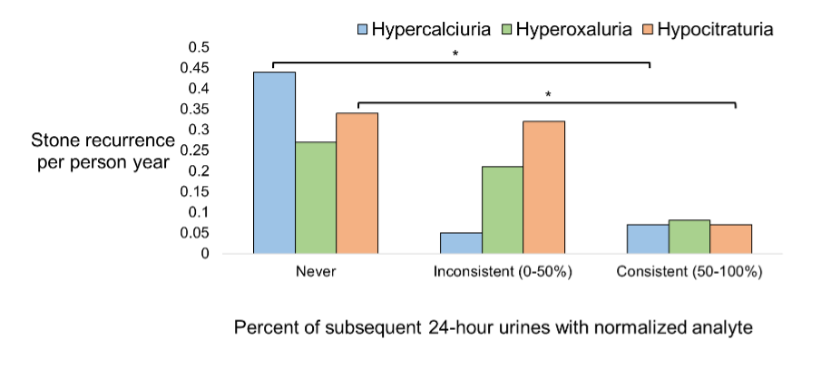

Supplement: Supplementary file 2 — Supplementary file2 Figure 2. Impact of 24-hour urine abnormality correction consistency on stone recurrence. *denotes statistically significant difference after multivariable adjustment (all p < 0.05) (PNG 53 KB) [file 240_2025_1910_MOESM2_ESM.png]
